# The Multifaceted Role of Heme in Cancer

**DOI:** 10.3389/fonc.2019.01540

**Published:** 2020-01-15

**Authors:** Veronica Fiorito, Deborah Chiabrando, Sara Petrillo, Francesca Bertino, Emanuela Tolosano

**Affiliations:** Department of Molecular Biotechnology and Health Sciences, Molecular Biotechnology Center, University of Torino, Turin, Italy

**Keywords:** heme, cancer, iron, metabolism, microenvironment (ME)

## Abstract

Heme, an iron-containing porphyrin, is of vital importance for cells due to its involvement in several biological processes, including oxygen transport, energy production and drug metabolism. Besides these vital functions, heme also bears toxic properties and, therefore, the amount of heme inside the cells must be tightly regulated. Similarly, heme intake from dietary sources is strictly controlled to meet body requirements. The multifaceted nature of heme renders it a best candidate molecule exploited/controlled by tumor cells in order to modulate their energetic metabolism, to interact with the microenvironment and to sustain proliferation and survival. The present review summarizes the literature on heme and cancer, emphasizing the importance to consider heme as a prominent player in different aspects of tumor onset and progression.

## Introduction

The onset and progression of cancer rely on the ability of tumor cells to channel different biological processes toward the promotion of cell proliferation, the escape from immunosurveillance and the resistance to drugs. Inorganic iron has been reported to play pivotal roles in several aspects related to cancer metabolic adaptation and tumor microenvironment reprogramming (1–3). Similarly, organic iron, in the form of the iron-containing porphyrin heme, is potentially a best candidate molecule exploited/controlled by tumor cells in order to modulate the energetic metabolism, to interact with the microenvironment and to sustain proliferation and survival. Heme bears a series of functions that are far beyond those mediated by its iron atom, including oxygen transport and storage, drug and steroid metabolism, transcriptional and translational regulation, signal transduction and microRNA processing (4, 5). In addition, heme synthesis is a cataplerotic pathway for the tricarboxylic acid cycle, as it consumes succynil-CoA. However, among the different metabolic processes that cancer cells can regulate to meet their specific demands, heme metabolism has been so far marginally studied, and frequently the importance of heme for cancer has been attributed to the iron atom contained in heme, rather than to specific functions mediated by the entire heme molecule itself. The present review will summarize the literature on heme and cancer highlighting both positive and negative effects of heme on cancer cells and on components of the tumor microenvironment.

## Dietary Heme and Cancer

Historically, the role of heme in cancer has been studied focusing on the effects mediated by exogenous dietary heme on the organism. These studies contributed to the current notion that dietary heme is a risk factor for cancer. Heme is an iron coordinating porphyrin contained predominantly in red and processed meat in the form of hemoglobin and myoglobin. Red meat refers to unprocessed mammalian muscle meat, while processed meat refers to meat that has been transformed through salting, curing, fermentation, smoking, or other processes to enhance flavor or improve preservation. Recently, the International Agency for Research on Cancer (IARC), following an assessment of over 800 studies performed world-wide, classified processed meat as group 1 “carcinogenic to humans” and fresh red meat as group 2A “probably carcinogenic to humans” (6). Conversely, no link between white meat and fish and cancer has been found (7–9). For this reason, heme has been proposed as the key molecule contributing to tumorigenesis upon red and processed meat intake.

The role of dietary heme in cancer has been highlighted in different types of carcinomas. Indeed, high consumption of red and processed meat has been associated with increased incidence of esophageal, gastric, breast, endometrial, pancreas and lung tumor (10–15), while no clear link was found for bladder and prostate cancer (16–18). However, the majority of studies focused on the role of dietary heme in the pathogenesis of colorectal cancer (CRC), still a leading cause of cancer deaths in Western Countries (19–21). Dietary heme is absorbed mostly in the upper part of the small intestine. Once absorbed, heme is degraded by the action of the enzymes heme oxygenases (HMOXs) into biliverdin, carbon monoxide (CO), and iron (Fe^2+^), that is then scavenged by the protein ferritin (4). However, if red/processed meat is assumed in large amounts, all the ingested heme cannot be absorbed by the small intestine and it accumulates for a considerable time in the large intestine (22, 23). In presence of high free heme levels both ferritin and HMOXs are saturated and cells accumulate free heme and labile iron that exert a variety of cytotoxic effects on intestinal mucosa (24). For example, heme is able to induce cytotoxic damage to surface epithelial cells that changes surface to crypt signaling, resulting in hyperproliferation and finally hyperplasia of crypt cells in heme-fed mice (25). Furthermore, free heme and labile iron accumulation result in the production of reactive oxygen species (ROS) that pathologically oxidize DNA, lipids and proteins. It has been well-demonstrated that ROS-induced DNA damage and gene mutations cause CRC and that proteins involved in CRC development are redox-sensitive (26). Additionally, ROS are able to induce lipid peroxidation of intestinal cells. Reactive lipid peroxides, formed by the action of ROS, covalently bind to the protoporphyrin ring of heme giving rise to an extremely lipophilic molecule, named cytotoxic heme factor (CHF) that induces cytotoxic damage on intestinal epithelial cells (27). To note, processed meat when in contact with gastric acid can also give rise to lipid hydroxiperoxide (LOOHs). LOOH is then broken down by the iron released by heme to produce free radicals and, subsequently, aldehyde molecules like malondialdehyde (MDA) and 4-hydroxynonenal (4-HNE) (28). MDA is toxic and it is able to bind DNA forming mutagenic adducts. 4-HNE induces apoptosis and kills normal cells, but not precancerous cells that are mutated on Adenomatous Polyposis Coli (APC) gene (29).

In addition to the processes described above, heme is also able to catalyze the formation of N-nitroso compounds (NOC) in the gastrointestinal tract (30). NOC are known carcinogens that can determine DNA mutation through alkylation, and high NOC concentrations have been associated to increased red meat consumption (31, 32). However, it is important to underline that NOC found after ingestion of red meat in humans consist mainly of nitrosyl iron and nitrosothiols, products that have profoundly different chemistries as compared to some tumorigenic N-nitroso species (33). Therefore, more studies are required to clarify the real involvement of heme in NOC pro-tumor effects.

Finally, it has been demonstrated that heme alters the normal intestinal bacterial flora especially by decreasing the number of gram-positive bacteria (34) leading to a state of dysbiosis (microbial imbalance or maladaptation) that exacerbates colitis and adenoma formation in mice (35) and is correlated to the insurgence of CRC (35–37). Moreover, the gut microbiota can induce *per se* hyperproliferation via mechanisms occurring in the colon lumen, such as modulation of oxidative and cytotoxic stress or by influencing the mucus barrier, and these effects are intensified in presence of heme (38). Indeed, a recent study showed that mice receiving a diet with heme show an increased population of mucolytic bacteria in their colon. These bacteria, synergistically with heme-produced CHF, damage gut epithelium and lead to a compensatory aberrant hyperproliferation. Conversely, mice receiving heme together with antibiotics do not show this phenotype (38).

**Figure 1:**
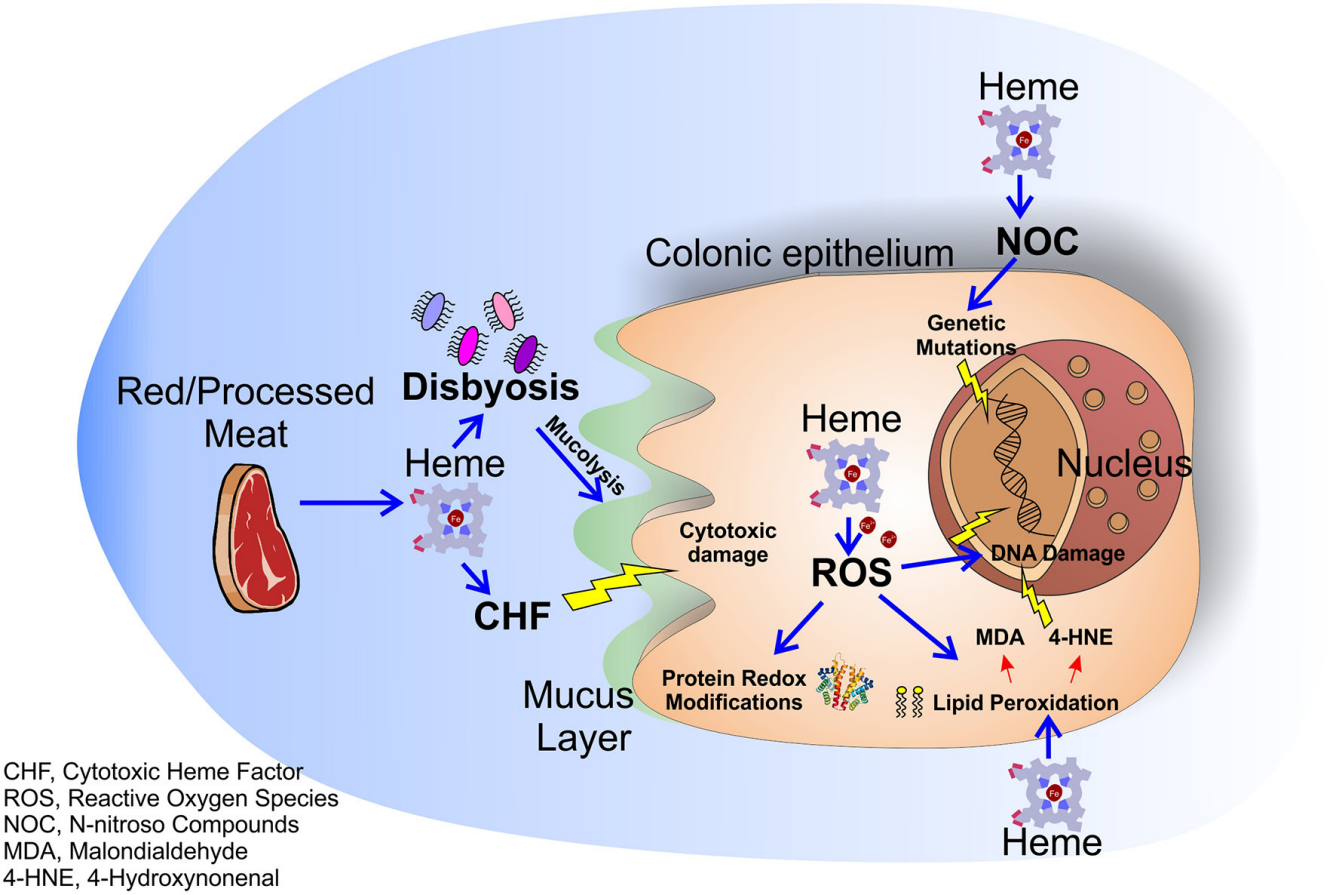
Molecular mechanisms of dietary heme induced colorectal carcinogenesis. Heme in red/processed meat alters multiple molecular and genetic mechanisms in the colonic epithelium resulting in colorectal carcinogenesis. Heme accumulation induces the formation of the CHF that leads to cytotoxic damage to surface epithelial cells. Moreover, heme causes peroxidation of lipids and NOC formation resulting in free radical formation and genetic mutations. Labile iron, resulting from heme degradation, induces the formation of ROS that lead to oxidative damage and genetic mutations. Finally, heme alters the intestinal flora enhancing the heme-induced cytotoxic effects.

Overall, the studies on dietary heme and cancer support the idea that heme contained in food can sustain cancer by different mechanisms (Figure 1). However, it must be recognized that methodologies employed in some of these studies have been challenged. Indeed, some animal studies took advantage of diets low in calcium and high in fat, combined with the exposure of heme frequently at doses higher than that expected with a normal dietary consumption of red meat. Moreover, pork meat shows low heme levels, but it has been associated with CRC. Finally, it cannot be excluded that carcinogenesis could be ascribed to other molecules contained mostly in red meat, not related to heme (33). Therefore, further research is required to clarify the role of heme present in red and processed meat in cancer.

## Heme Synthesis and Cancer

Heme can be acquired by dietary sources, but in addition, all the cells in the organism are able to synthesize heme. Heme synthesis includes eight different reactions occurring partly in mitochondria and partly in the cytosol. The first rate limiting step is based on the condensation of succynil-CoA and glycine to produce 5-aminolevulinic acid (ALA), a reaction catalyzed by the enzyme 5-Amilolevulinate Synthase 1 (ALAS1). Then, by subsequent reactions involving the enzymes ALA dehydratase (ALAD), porphobilinogen deaminase (PBGD), uroporphyrinogen III synthase (UROS), uroporphyrinogen decarboxylase (UROD), coproporphyrinogen oxidase (CPOX), protoporphyrinogen oxidase (PPOX) and ferrochelatase (FECH), heme is finally produced (4, 39).

The study of heme synthesis in tumors has raised interest since many years. Indeed, in nineties, it was discovered that tumors, upon ALA administration, are able to accumulate remarkably higher amount of protoporphyrin IX (PpIX) as compared to normal tissues, and this property was demonstrated to be exploitable for tumor fluorescence-guided surgery (FGS) and to kill cancer cells by photodynamic therapy (PDT) (40–42). Since then, extensive research has been performed to determine the molecular mechanism involved in enhanced ALA-PpIX accumulation in tumor cells. Particularly, the rate of heme biosynthesis in different kinds of tumor was dissected in several works, leading to accumulation of conflicting results. However, ALAS1, PBGD and UROD expression and/or activity were frequently found up-regulated in cancer (43). Consistently, repression of heme biosynthesis by the ALAD inhibitor succinylacetone was shown to reduce tumor cell survival and proliferation (44–46). Conversely, FECH levels were found often down-modulated in tumor cells as compared to normal cells (43, 47).

**Figure 2:**
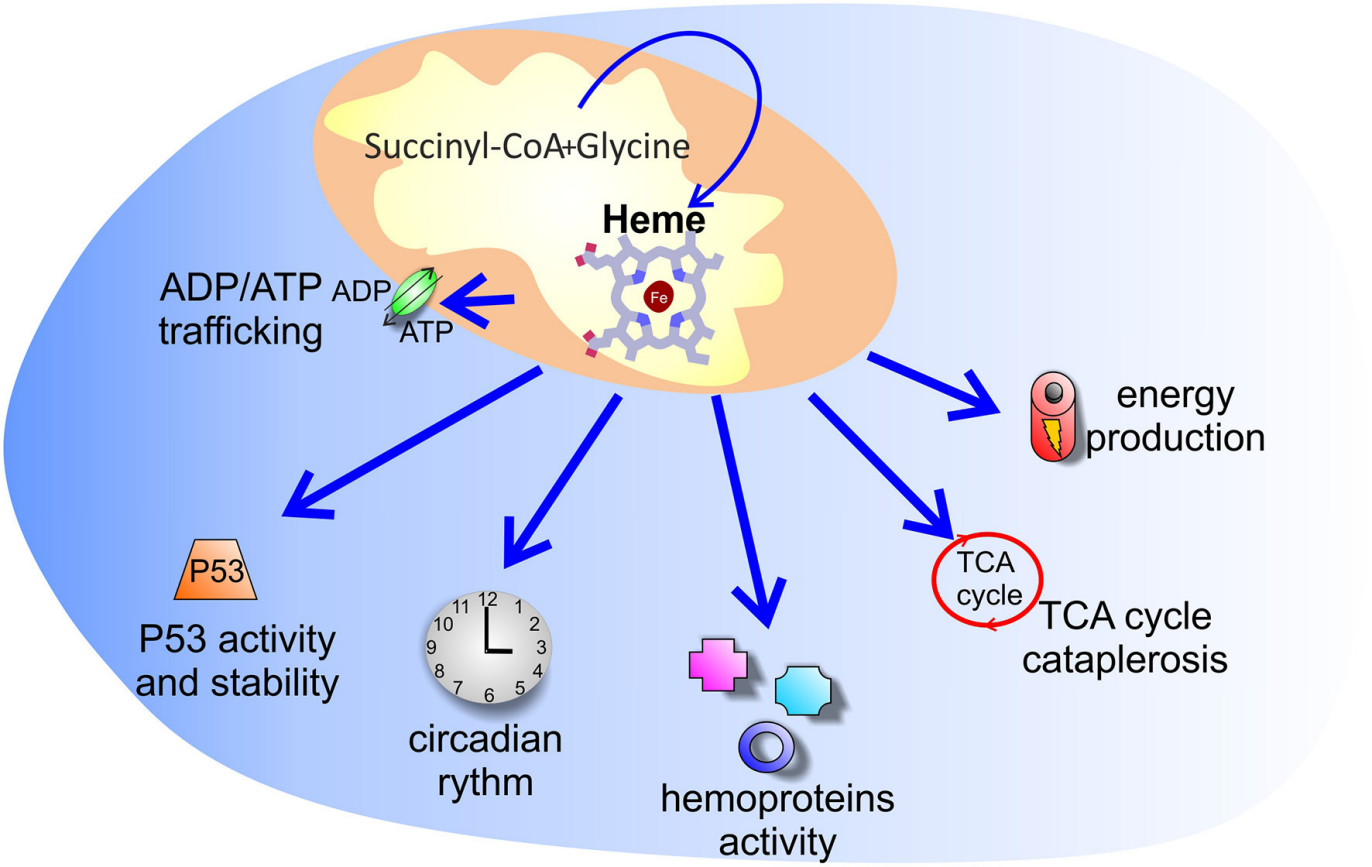
Putative processes controlled by increased heme synthesis in cancer. The reason why tumors enhance heme synthesis is unknown. Several hypotheses have been explored: increased heme synthesis could control energy production, ADP/ATP exchange between mitochondria and cytosol, TCA cycle cataplerosis, P53 activity and stability, as well as the activity of hemoproteins and of heme binding proteins involved in the circadian clock machinery.

Taking together these discoveries, it can be concluded that tumors are characterized by high porphyrins synthesis, not necessarily associated to final heme production. This conclusion, however, is controversial. Indeed, cancer cells have been shown to display high heme levels (45, 48), increased activity of heme containing proteins (45, 46, 49) and enhanced expression of heme exporters (45, 50), suggesting that heme, and not only its precursors, is produced and that the entire heme biosynthetic pathway is promoted in tumors. Therefore, the reason why tumors accumulate more ALA-mediated PpIX than surrounding normal tissues and why tumors enhance heme synthesis remains a fundamental question to be answered (Figure 2).

Considering the fact that heme is a crucial cofactor for complexes of the electron transport chain (ETC), one possible explanation for increased heme biosynthesis in tumors is that it could be exploited by cells to sustain oxidative phosphorylation (OXPHOS). Indeed, despite the well-accepted model proposed by Otto Warburg and co-workers, pointing to increased glycolysis in cancer cells, many lines of experimental evidence have shown that the function of mitochondrial OXPHOS in most tumors is intact and that the vast majority of tumor cells generate adenosine triphosphate (ATP) via oxidative phosphorylation (51). Data obtained on the human lung carcinoma cell line A549 showed that induction of heme biosynthesis by ALA enhances OXPHOS in these cells (52). Similarly, in additional studies on lung cancer (46) and in an *in vitro* model of myeloid leukemia (45), it was demonstrated that increased heme synthesis is associated to higher oxygen consumption, an indicator of OXPHOS, and the effect was prevented by cell treatment with succynilacetone.

Nevertheless, tumors like hereditary leiomyomatosis and renal-cell cancer (HLRCC), due to mutations in the tricarboxylic acid (TCA) cycle enzyme fumarate hydratase, show reduced OXPHOS and sustained glycolysis but increased heme synthesis (53). Similarly, human colon carcinomas overexpress the mitochondrial ATPase Inhibitory Factor 1 (ATPIF1), an inhibitor of the mitochondrial H^+^-ATP synthase (54) and OXPHOS, as well as a promoter of iron incorporation into PpIX by FECH (55). These studies support the idea that suppression of OXPHOS and enhanced glycolysis in tumors could be associated to increased heme biosynthesis, suggesting that heme can mediate additional functions in cancer, unrelated to its role as a cofactor for ETC complexes.

A complex interplay between heme and energy metabolism is further supported by studies indicating that ALAS1 expression is suppressed by glucose (56). Moreover, it has been recently reported that heme can negatively regulate the activity of pyruvate dehydrogenase (PDH) by binding the PDHA1 subunit, favoring a switch from pyruvate oxidation in the mitochondria to its glycolytic conversion into lactate (57). In addition, heme can control the trafficking of ADP and ATP between mitochondria and cytosol. The translocase involved in the ADP/ATP exchange is the adenine nucleotide transporter (ANT), an integral protein located in the inner mitochondrial membrane. While in rodents only three ANTs exist (ANT1, 2, and 4), in humans there are four ANT isoforms (ANT1, 2, 3, and 4), encoded by four distinct genes with different promoter sequences, supporting distinct regulatory control. In humans, ANT1 is mainly expressed in heart, skeletal muscle and brain, while ANT3 is ubiquitous (58) and ANT4 is expressed in testis and germ cells (58). Conversely, ANT2 is poorly detectable in tissues, but its expression can dramatically increase in cancer and proliferating cells with high glycolytic rates and/or low oxygen (58, 59). ANT1 and ANT3 export ATP synthesized in mitochondria toward the cytosol, while several evidences suggest that ANT2 may realize an inverse exchange, translocating the glycolytic ATP synthesized in the cytosol toward the mitochondrial matrix (59). Moreover, ANT1 and 3 are crucial component of the mitochondrial permeability transition pore complex and play a major role in mitochondria-mediated cell death (60–62). Heme binds ANT1, 2, and 3 isoforms and ANTs are believed to contribute to heme biosynthesis by transporting heme precursors into mitochondria (63). Particularly for ANT1, it has been demonstrated that heme binds to the center pore domain and to the residues that associate to ADP, and binding of heme, or of the heme precursors PpIX and coproporphyrin III, inhibits the ADP uptake in a competitive manner (63). Moreover, heme can transcriptionally repress the expression of ATP/ADP carrier (AAC) 3, the yeast ANT2 orthologous, through the ROX1 factor (64). Interestingly, human ANT2 promoter shows a negative regulatory motif, called glycolysis regulated box (GRBOX) that is a ROX1-like motif (59), suggesting possible heme-dependent regulation also of human ANT2. This could be particularly relevant in cancer, because ANT2 has been postulated to contribute to the rapid metabolic adaptation of tumor cells to glycolysis and hypoxic stress by preserving the mitochondrial ATP content necessary to maintain the mitochondrial membrane potential and intra-mitochondrial metabolic pathways when OXPHOS is impaired (58, 65). Moreover, additional studies have highlighted an anti-apoptotic role for ANT2 (66), its implication in the PI3K/Akt pathway (67), frequently activated in cancer, and in the regulation of cancer-related microRNAs (67). Finally, ANT2 is considered a promising therapeutic target to control tumor cell growth, migration, invasion and chemosensitization (68). Therefore, the modulation of heme synthesis, by affecting ANTs activity and expression, could have consequences on the transport of both OXPHOS-derived ATP from mitochondria to cytosol or of glycolytic ATP from the cytosol to the mitochondria.

Overall, the role of heme synthesis in the modulation of cancer energy metabolism remains controversial.

Another possibility is that increased heme synthesis in tumors is intended to support the activity of specific hemoproteins. Cells are equipped with several hemoproteins (69) and for some of them a role in cancer has been reported, including myoglobin (70), tryptophan 2,3-dioxygenase (TDO) and indoleamine-2,3-dioxygenase 1 and 2 (IDO1/2) (71–73), mitochondrial cytochromes (74, 75), cytochrome P450 (76, 77), and cyclooxygenases (78, 79). Studies on lung cancer demonstrated that the amount of oxygen-utilizing hemoproteins, such as cytoglobin, cytochrome c, cytochrome P450 family 1 subfamily B member 1 (CYP1B1) and prostaglandin-endoperoxide synthase 2 (COX2) are higher in tumor cells as compared to normal cells and that, at least for cytoglobin and cytochrome c, their levels depend on the rate of heme synthesis and cellular heme content (46). A similar phenotype was observed in human non-small cell lung cancer (NSCLC) tissues (49). Moreover, in colorectal cancer cells ALA administration results in the down-modulation of cyclooxygenase 2 expression, although its overall enzymatic activity is maintained (80). These hemoproteins can control oxygen availability or participate in crucial metabolic processes tightly modulated in cancer cells to allow/promote cell survival and proliferation, as well as to escape tumor immunosurveillance. Furthermore, heme can interact and regulate the activity of additional proteins with an already reported role in cancer. For example, heme is a crucial regulator of the nuclear receptor subfamily 1 group D member 1 (NR1D1 or Rev-erbα) (81), as well as an interactor for neuronal PAS domain protein 2 (NPAS2) (82) and period circadian regulator 2 (PER2) (83, 84). All these proteins are involved in the circadian clock mechanism, and alterations in circadian rhythm is typically observed in cancer, so that some of the clock machinery components have been proposed as targets in cancer therapy (85). Therefore, heme synthesis promotion in tumor cells could also partly contribute to modulate this system in order to sustain cell growth.

In addition, increased heme synthesis in cancer cells may be promoted in order to regulate the tumor suppressor P53, a transcription factor that controls a broad and flexible network of biological processes, including DNA damage response, autophagy, cellular metabolism, epigenetics, inflammation, just to cite the most relevant ones (86). Interestingly, Shen et al. (87) demonstrated that the stability of P53 is directly regulated by heme. Indeed, heme binds to a C-terminal heme-responsive motif (HRM) of P53. The heme-P53 interaction interferes with the P53 DNA binding activity *in vitro*, thus suggesting that heme may modulate the transcription of P53 target genes. Furthermore, the binding of heme to P53 promotes its nuclear export and degradation via the ubiquitin-proteasome system. These findings provide mechanistic insights into the process of tumorigenesis associated with iron excess. It is well-established that iron excess is a hallmark of several tumor types (88). A positive correlation between iron and heme levels *in vivo* has been reported (87), leading to the hypothesis that iron excess in cancer sustains the synthesis of heme, that in turn directly affects P53 stability and function. Considering the increasing amount of data showing that tumors reprogram heme metabolism (not only iron metabolism) to achieve advantages in terms of proliferation and survival, it is tempting to propose that the alteration of heme metabolism that occurs during tumor onset and cancer progression may contribute to the dysregulation of P53 expression.

In the end, it has been proposed that heme synthesis is enhanced in cancer in order to mediate TCA cycle cataplerosis. By this view, enhanced heme biosynthesis is primarily intended to consume succynil-CoA rather than to produce heme. In HLRCC it has been demonstrated that increased heme biosynthesis is crucial to avoid the accumulation of toxic TCA cycle intermediates, as a consequence of mutations in fumarate hydratase. Heme produced in this system is then addressed to degradation by the enzyme HMOX1, indicating that its production is not intended to increase the intracellular bioavailable heme pool (53).

Overall, it seems clear that heme biosynthesis is frequently enhanced in tumors and that this phenomenon could serve different and sometimes conflicting purposes. Additional studies are required to fully elucidate the underlying mechanism that can reconcile all these aspects.

## Heme Import/Export/Degradation and Cancer

Other than being synthesized, heme can also be imported inside the cell by three main importers: (1) the solute carrier family 46 member 1 (SLC46A1 or HCP1), a folate importer able to transport also heme, (2) the feline leukemia virus subgroup C receptor family member 2 (FLVCR2), and (3) the solute carrier family 48 member 1 (SLC48A1 or HRG1). Among them, FLVCR2 and HCP1 are exposed on the cell plasma membrane, while HRG1 is localized on endolysosomes.

The role of FLVCR2 in normal cells is not well-studied and to our knowledge there is only one paper to date in which FLVCR2 was analyzed in cancer. In this paper, interestingly, it was observed that FLVCR2 is overexpressed in bovine papillomavirus-associated urinary bladder cancer (89).

Regarding HCP1 and HRG1, their levels were reported to be dramatically increased in lung cancer cells/tissues (46, 49), where they contribute to import heme in order to ensure proper activity of hemoproteins. Indeed, depletion of heme in the culture medium of tumor cells or the use of heme-sequestering peptides, to avoid heme uptake, result in the down-modulation of hemoproteins activity (46, 49). In addition, high expression of HCP1 was detected in gastric cancer cells as compared to normal gastric cells (90). Moreover, HRG1 overexpression was observed in HeLa cells and in highly invasive and migratory cancer cell lines, where it can be detected not only in endolysosomes but also on the cell plasma membrane. Its silencing in these cells was demonstrated to result in reduced survival and migratory capacity, while the opposite phenotype was observed upon its over-expression (91, 92). In HeLa cells it was demonstrated that HRG1 interacts with the vacuolar H^+^-ATPase and regulates its activity, thus modulating the acidification of endosomes (91). By this way, HRG1 has a unique role in regulating pH-dependent endocytic pathway, with an impact on the ability of the cells to acquire nutrients, to mediate signaling in response to growth factor receptor activation, and to internalize and traffic integrins and other proteins, which is necessary for cell survival, migration, and proliferation (91). Moreover, in additional highly invasive and migratory cancer cell lines, it was shown that, by the interaction with the vacuolar H^+^-ATPase, HRG1 participates to the regulation of cytosolic/extracellular pH gradient. Indeed, HRG1 indirectly favors the alkalinisation of cell cytosol and the acidification of the extracellular environment, a condition that enhances extracellular matrix degrading enzymes expression and activity, facilitating a more invasive phenotype of cancer cells (92). In addition HRG1 expression in these cells was associated to a pH-dependent promotion of glucose transporter 1 (GLUT1) trafficking to the plasma membrane, leading to increased glucose uptake and glycolysis, thus favoring cell growth (92).

Altogether, these studies indicate that heme import is enhanced in cancer with the aim to promote the activity of specific hemoproteins and to modulate pH gradient and cell metabolism.

Together with heme import, also heme export has been shown to be increased in cancer. Heme export is mediated by the specific heme exporter feline leukemia virus subgroup C receptor family member 1 (FLVCR1). Moreover, the ATP binding cassette subfamily G member 2 (ABCG2), a known exporter for a broad range of molecules and xenobiotics, is also involved in heme/porphyrins export.

The expression of FLVCR1 has been reported up-regulated in different kinds of tumors, including bovine papillomavirus-associated urinary bladder cancer (89), synovial sarcoma (50), and hepatocellular carcinoma (93). For hepatocellular carcinoma, the high expression of FLVCR1 was associated to higher neoplasm disease staging, adjacent tissue inflammation, vascular invasion and neoplasm histologic grade, as well as to reduced overall survival and disease-free status (93). In synovial sarcoma cells, the silencing of FLVCR1 was associated to reduced cell proliferation, survival and tumorigenicity *in vitro* and *in vivo* (50). Similarly, FLVCR1 silencing impairs the survival of neuroblastoma cells *in vitro*, particularly upon ALA administration (94).

The literature on ABCG2 and cancer is very rich; however, it is important to distinguish between its role as a heme/porphyrins exporter as compared to its role in the efflux of additional unrelated substrates and xenobiotics. Focusing on heme, it has been shown that triple negative breast cancer cell lines have significantly reduced ALA-PpIX levels as compared with estrogen receptor (ER) positive and human epidermal growth factor receptor 2 (HER2) positive breast cancer cell lines because of elevated ABCG2 activity (95). In line with this, high ABCG2 expression was considered a major cause of failure of ALA-photodynamic therapy in different kinds of tumors, as it can induce resistance to this kind of treatment by preventing the accumulation of the photosensitizer PpIX inside the cells (96, 97). Moreover, in an *in vitro* model of myeloid leukemia, it has been shown that MYCN drives increased heme synthesis and that, in this system, the excess of PpIX produced is promptly exported out of the cells by ABCG2 to avoid cell toxicity (45). Therefore, in these cells the forced stimulation of heme synthesis, associated with the imposed reduction of ABCG2 expression, leads to tumor cell death.

Overall, the literature on heme import and export in cancer looks counterintuitive, indicating that both processes are enhanced in tumor cells. Although an explanation for these apparently conflicting phenomena does not exist, it could be postulated that the two systems are both promoted in order to establish a new equilibrium in heme homeostasis. Another possibility is that import and export target two different pools of heme, and that cellular heme compartmentalization could play a role in this context. Interestingly, it has been shown that heme export by FLVCR1 is highly associated to heme synthesis (98), thus supporting the idea that the trafficking of endogenously synthesized heme could be managed differently as compared to exogenous heme imported into the cells. Additional studies are required to fully elucidate the biological significance for these alterations of heme trafficking in cancer.

Finally, intracellular heme homeostasis benefits of an additional system to control heme levels, which is the degradation of the molecule. As described above, HMOXs, the rate-limiting enzymes in heme catabolism, catalyze the stereospecific degradation of heme to biliverdin, with the concurrent release of ferrous iron ions and CO. There are two main HMOXs isoforms, encoded by two distinct genes: HMOX1 and HMOX2. HMOX2 is a constitutively expressed protein, while HMOX1 can be strongly induced in many tissues in response to cellular stress caused by a wide spectrum of stimuli including, but not restricted to, heme. The literature on HMOXs and cancer is wide, and a comprehensive summary of works on this topic can be found in several reviews (99–101). Nevertheless, also in this case it is important to distinguish between HMOXs role as heme degrading enzymes as compared to their non-catalytic role. Indeed, it has been shown that enzymatically inactive HMOX1 could translocate to the cell nucleus and exerts gene expression regulatory functions (102, 103). The role of HMOX1 in tumor cell proliferation is controversial: its expression in tumors was found very frequently up-regulated and a role for HMOX1 as a mediator of ROS-promoted cell proliferation was established; on the other hand, however, in other kinds of tumors an HMOX1 anti-proliferative action was demonstrated, often associated to effects mediated by CO and biliverdin (99, 101). However, most experiments support a permissive role of HMOX1 in tumor growth. What seems to be clear is that HMOX1 can affect different aspects of tumorigenesis, encompassing regulation of cell proliferation and differentiation, promotion of cytoprotection and inhibition of apoptosis, induction of angiogenesis and metastatization, as well as immunosuppression (99–101). Moreover, HMOX1 activity may affect anti-tumor therapies, as its expression is further elevated in response to radio-, chemo-, or photodynamic therapy and is involved in resistance to them (100, 101). Summarizing, the expression of HMOX1 and the activity of its byproducts can provide the selective advantage for tumor cells to overcome the increased oxidative stress occurring during tumorigenesis and/or as a consequence of anti-tumor therapies.

## Heme and Tumor Microenvironment

**Figure 3:**
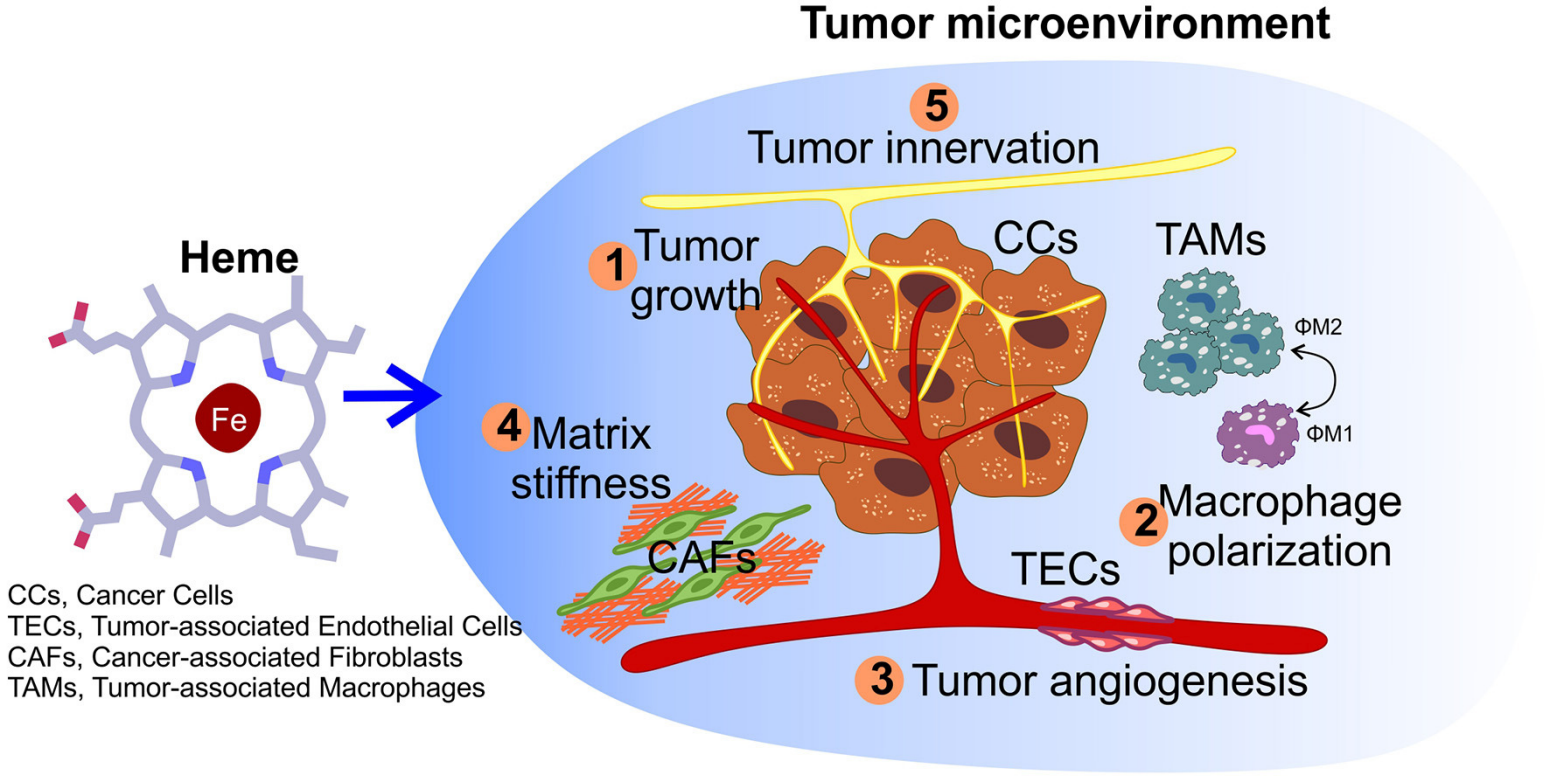
Impact of heme on tumor microenvironment. Other than sustaining cancer cells growth (1), heme could also potentially affect other processes within the microenvironment, including macrophage polarization (2), the angiogenic potential of tumor endothelial cells (3), matrix remodeling by cancer-associated fibroblasts (4), and nerve-cancer cross-talk (5).

Other than acting directly on tumor cells, heme can exerts its functions in cancer by modulating the tumor microenvironment (TME) (Figure 3). The TME is composed by tumor cells, endothelial cells of surrounding blood vessels, myofibroblasts, stellate cells, adipose cells, peritumor nerve cells, immune cells, endocrine cells, fibroblasts, and the extracellular matrix (104, 105). All the cell types in the TME contribute to tumor progression mainly by releasing factors, which establish a favorable environment for the cancer cell and promote tumor cell survival and migration, metastasis formation, chemo-resistance, and the ability to evade the immune system responses (104).

Heme has been reported to modulate the activity of tumor-associated macrophages (TAMs). Macrophages have heterogeneous phenotypes that range from the “classically-activated” pro-inflammatory M1-cells to the “alternatively-activated” anti-inflammatory M2-cells (106). If on one hand M1-like macrophages have anti-tumor properties, on the other hand macrophage polarization toward a M2-like phenotype correlates with pro-tumor activities, such as enhanced angiogenesis, matrix remodeling, and immune suppression (107, 108). Due to the specific cues that characterize the tumor microenvironment, TAMs are induced to preferentially acquire a M2-like specialized phenotype that protects cancer cells from targeted immune responses (109). Notably, M. Costa da Silva and colleagues demonstrated that TAMs exposed to haemolytic red blood cells (RBCs), a condition sometimes observed in cancer due to the extravasation of RBCs from the abnormal tumor-associated vessels (110), accumulate iron intracellularly and acquire a M1 pro-inflammatory phenotype, which in turn promotes tumor cell death (111). This is in line with studies on haemolytic diseases, such as sickle cell disease, where it has been observed that macrophage exposure to free heme, released by damaged erythrocytes, leads to M1-reprogramming (112). Moreover, these findings are in agreement with additional studies reporting the concept that iron can drive macrophages polarization toward a pro-inflammatory M1-phenotype (113, 114). The effects on TAMs described can be mainly ascribed to the iron atom contained in heme. However, another important aspect that should be taken into account is the role exerted by CO in the control of myeloid cell differentiation. Indeed, the CO produced upon heme degradation by HMOXs has been reported to cause tumor regression and increased drug sensitivity in prostate and lung cancer models (115), and this can be partly attributed to CO effects on macrophages within the TME. Specifically, it has been shown that CO treatment in mice leads to an increased number of M1-like macrophages and reduced tumor growth (116). Taken together, these works have led to the implementation of strategies able to modulate the exposure of macrophages to heme or iron, as the use of iron oxide nanoparticles (109, 111), for cancer therapy.

Another fundamental component of the tumor microenvironment is represented by tumor-associated endothelial cells (TECs). TECs strongly differ from their normal counterparts (117) as they display a high pro-angiogenic potential that mostly relies on their enhanced ability to proliferate and migrate. The switch of TECs from quiescence to a highly active state is favored by a specific metabolic reprogramming (118–120). The cross-talk between cancer cells and TECs promotes aberrant neo-angiogenesis, which is required to sustain tumor growth, by providing oxygen and nutrients, and to favor metastatization (117, 121, 122). To our knowledge, there are no studies in literature analyzing the role of heme in TECs. However, we demonstrated that alterations in endothelial intracellular heme metabolism strongly affect the angiogenic process during development (98, 123). Consistently, another study highlighted the critical role of the heme biosynthetic pathway in supporting endothelial functions (124). In addition, tumor angiogenesis is also promoted by the increased stiffness of the extracellular matrix (ECM) found in TME (125, 126). High ECM stiffness is mainly due to increased collagen deposition and increased cross-linking within the tumor stroma. Matrix remodeling is primarily controlled by the activity of a group of zinc-dependent endopeptidases, named matrix metalloproteinases (MMPs), which are mainly released by cancer-associated fibroblasts (CAFs). However, recent studies highlighted the involvement in this process of peroxidases released by immune cells, such as myeloperoxidase (MPO) and eosinophil peroxidase (EPO). In particular, MPOs and EPOs have been reported to be able to directly induce the secretion of collagen I and collagen VI by CAFs (127), thus increasing matrix stiffness and promoting tumor angiogenesis and metastatization (128). Notably, MPOs and EPOs are heme-containing enzymes, thus suggesting that changes in the amount of available heme within the cells could affect the activity of these enzymes in matrix remodeling. Taking together all these considerations, it is tempting to speculate that heme could affect different processes involved in tumor angiogenesis and future studies will help to verify this hypothesis.

Finally, an additional promising aspect to dissect in the future is the possible implication of heme in the control of tumor innervation. The peripheral nervous system is nowadays gaining growing interest in cancer research due to its role in modulating both cancer cells and TME. This is achieved through the reciprocal interaction between nerves and cancer cells (nerve-cancer cell cross-talk), as well as between nerves and the TME (129–131). Indeed, recent data clearly indicate that tumor onset and progression is accompanied by increased innervation, through a mechanism largely dependent on the secretion of neurotrophic factors by cancer cells. Furthermore, nerves influence tumor onset, progression and metastasis formation, mainly through the secretion of neuropeptides and neurotransmitters in TME, where they interact with receptors expressed by cancer cells and by other cells of the TME (131–136). Heme is required for the survival of different types of neuronal cells (137, 138); however, the specific role of heme in the peripheral nervous system and its potential implication in tumor innervation is completely unknown. Several evidences suggest that heme is crucial for the maintenance of the peripheral nervous system, particularly for sensory neurons, one of the types of nerves that actively innervates tumors (139–141). Notably, mutations in genes encoding proteins involved in heme synthesis and export have been reported in diseases characterized by the degeneration of sensory neurons (94, 142–145). Finally, heme may also regulates pathways important for nerve-cancer cell cross-talk. Indeed, heme is involved in the regulation of gene expression in neurons via nerve growth factor (NGF) signaling (146), thus suggesting that heme may modulate nerve outgrowth in the tumor microenvironment. Furthermore, heme also regulates the metabolism of some neurosteroids and neurotransmitters (137), with a potential implication in nerve-cancer cross-talk. In addition, other than sustaining cancer, tumor innervation is also one of the main cause of chronic pain in oncologic patients, particularly those in the advanced stage of the disease (147, 148). Interestingly, mutations in the heme exporter *FLVCR1* have been reported in patients with peripheral sensory neuropathy (94, 149, 150), thus indicating an involvement of heme in pain perception. Furthermore, CO produced upon heme catabolism can act as an atypical neurotransmitter or neuromodulator in the nervous system and is involved in nociception regulation (151). Overall, these evidences support the idea that heme could be implicated in different aspects of tumor innervation. Future targeted *in vitro* and *in vivo* experiments will definitively verify whether this hypothesis is correct.

## Additional Heme Functions Potentially Relevant for Cancer

In the previous paragraphs, we discussed the works that contributed to explore the role of heme in tumor growth and metastatization. However, we believe that heme could be involved in additional aspects of tumor biology, not investigated so far. Indeed, emerging evidences indicate that heme is required for the processing of microRNA (miRNA) that, by regulating more than 50% of the mammalian genome are implicated in several pathways crucial for cancer onset, growth and metastatization (152). Specifically, the RNA-binding protein DiGeorge critical region-8 (DGCR8), which is essential for the first processing step of pri-miRNAs, is a heme-binding protein (153–156). Heme binding to DGCR8 is required for its dimerization and activation (153) and the modulation of heme availability was reported to affect pri-miRNA processing *in vitro* (157). A correlation between alterations of heme and miRNAs expression was also reported. For instance, ALA-mediated sonodynamic therapy or PDT is associated with the altered expression of selected miRNAs (158–161). Although, further studies are required to fully understand the physio-pathological implications of these findings, these data suggest that mysregulation of miRNAs may represent an additional mechanism through which alterations of heme metabolism sustain and promote cancer progression.

In addition, several data support a potential involvement of heme in epigenetic modifications, that control multiple processes essential for cancer cells (162, 163). This is suggested by the observation that heme regulates the transcriptional and demethylase activity of the yeast histone demethylase GIS1 (GIS1), that belongs to the lysine demethylase 4 (jmjd-2/KDM4) subfamily of demethylases implicated in histone methylation, cellular signaling and tumorigenesis (164). The yeast GIS1 protein is conserved from yeast to mammals, suggesting a possible role for heme in the regulation of this protein also in mammals. Furthermore, several heme-regulated proteins of the circadian rhythms machinery are epigenetic modifying enzymes themself. For instance, clock circadian regulator (CLOCK) is a histone acetyltransferase and Rev-erbα functions as a transcriptional repressor by forming a complex with the nuclear receptor corepressor (NCOR) and the histone deacetylase 3 (HDAC3) (165). Finally, the activity of epigenetic modifying enzymes relies on the availability of specific metabolites (like α-ketoglutarate) and cofactors (acetyl-CoA, S-adenosylmethionine, and nicotinamide adenine dinucleotide). Most of them are produced by the TCA cycle. Since heme biosynthesis is a TCA cycle cataplerotic pathway (166), it is reasonable that alterations of heme homeostasis may affect the availability of metabolites to epigenetic modifying enzymes. Based on the role of heme in the control of cellular metabolism (166) and circadian rhythms (81), we propose that the alterations of heme metabolism observed in cancer may also contribute to the mysregulation of cancer epigenetics.

Finally, heme metabolism has been reported as an apoptosis modifying pathway in acute myeloid leukemia (AML) (167). This is relevant, because it suggests the possibility to target heme metabolism in order to increase drug sensitivity in cancer cells. In addition, we observed that the modulation of heme metabolism induces paraptosis (123), at least in endothelial cells. This suggests the possibility to potentially exploit the regulation of heme metabolism in cancer therapy, as the availability of compounds inducing alternative forms of programmed cell death could be very useful to counteract the resistance to apoptosis in tumors (168). Similarly, the identification of heme as an inhibitor of the proteasome (169) appears as a promising property to be exploited for therapeutic purposes, particularly considering that proteosome inhibitors are successfully currently used in cancer therapy (170).

## Conclusions

By the present review, we attempted to provide a comprehensive overview of the literature on heme in cancer, highlighting heme participation in multiple processes that sustain tumor growth and metastatization, encompassing the control of mitochondrial metabolism, the function of hemoproteins and P53 signaling.

**Figure 4:**
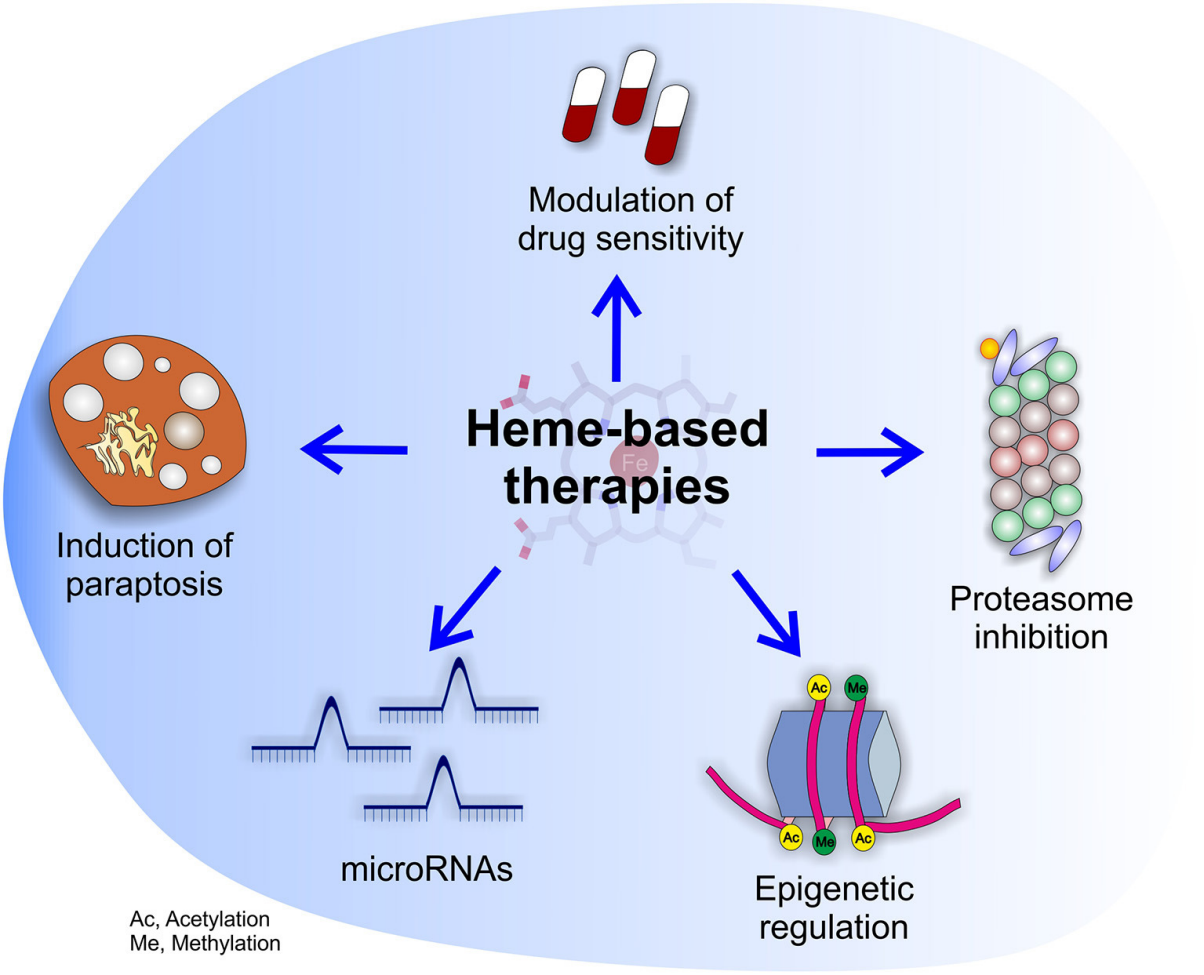
Potential effects of heme-based therapies. The multifaceted role of heme in cancer suggests the possibility to develop novel therapies targeting heme metabolism. The picture highlights the potential beneficial effects of heme-based therapies on cancer cells as well as tumor microenvironment.

Summarizing, the studies on dietary heme and cancer, although affected by some limitations, support the idea that heme contained in food can sustain cancer by different mechanisms. Conversely, the impact of endogenous heme in cancer is much more complex to envisage. On the one hand, heme biosynthesis is frequently enhanced in tumors, but this phenomenon could serve different and sometimes conflicting purposes. Moreover, both heme import and export are increased in tumor cells, but the reason is unclear. In addition, heme degradation by HMOX1 in tumor cells seems to support cancer by counteracting oxidative stress during tumorigenesis and upon anti-tumor therapies, but concomitantly to promote TAMs acquisition of a M1-like phenotype, favoring tumor regression and increased drug sensitivity. Finally, heme-containing enzymes like MPOs and EPOs can promote tumor angiogenesis and metastatization, and heme could also potentially affect cancer epigenetics, miRNAs and tumor innervation. Therefore, targeting heme metabolism is promising because it could have a broad impact on different aspects of cancer. For this reason, we envision that future work should be directed to the development of novel therapeutic strategies based on heme (Figure 4). However, the choice of the appropriate strategy is challenging, due to possible conflicting effects obtained by the block or the promotion of heme-related processes. To overcome these problems, further work is required in order to classify the precise tumor subtypes that can benefit of each single strategy.

Anyhow, according to present literature, the targeting of heme metabolism has already been exploited for cancer therapy. In particular, the stimulation of heme synthesis with ALA has been widely used for PDT (171). In addition, the indirect inhibition of heme synthesis through iron chelation therapy has been recently proposed for selected cancer types. Iron chelation therapy has emerged as an important chemotherapeutic strategy, because of the strong link between iron excess and tumorigenesis. However, iron-deprivation therapy was successful only on selected tumor types. The discovery that heme directly regulates P53 stability (87) explained the selective therapeutic efficacy of iron deprivation-based chemotherapy. Indeed, Shen et al. demonstrated that this selectivity was due to the P53 status of the tumor types (87). Specifically, iron chelation therapy, by decreasing heme levels, leads to the stabilization of P53 proteins only in tumors with wild-type P53, and not in case of *P53* mutations. As already suggested by Shen et al., these findings will allow the discrimination of the types of tumor that should benefit of iron chelation therapy (87). The targeting of heme synthesis through the deprivation of iron required for heme synthesis (iron chelation therapy) remains the best therapeutic strategy to date. However, because iron is essential for multiple processes beyond heme synthesis, a key challenge of chelation therapy is to balance iron levels in order to avoid excessive iron chelation. Moreover, it has to be underlined that emerging studies demonstrate that some kinds of tumor could be counteracted by iron supplementation, rather than by iron chelation therapy (172). Therefore, more specific strategies to blunt heme synthesis are required, not based on iron. The discovery that the beneficial effects of iron-chelation therapy on the growth of certain tumor types depends on the regulation of P53 by heme raises the possibility to directly target heme synthesis to counteract tumor growth. Several compounds, like succinylacetone or N-methyl protoporphyrin, are used to inhibit heme synthesis *in vitro*. Although these compounds are not yet used in the clinic, we cannot exclude that new drugs based on them will be developed. Similarly, it could be possible that, in the future, drugs aimed at blocking/stimulating heme importer/exporter/degrading proteins will be identified, in order to perturb heme-related mechanisms in cancer.

In conclusion, we hope that, in the next future, the growing awareness on heme role in processes relevant for cancer will stimulate research aimed at implementing innovative therapeutic approaches and at identifying the tumor subtypes sensitive to these treatments.

## Author Contributions

VF, DC, SP, and FB wrote the manuscript. ET critically revised the manuscript intellectual content.

### Conflict of Interest

The authors declare that the research was conducted in the absence of any commercial or financial relationships that could be construed as a potential conflict of interest.

## Abbreviations

IARC: International Agency for Research on Cancer
CRC: colorectal cancer
HMOXs: heme oxygenases
CO: carbon monoxide
Fe^2+^: ferrous iron
ROS: reactive oxygen species
CHF: cytotoxic heme factor
LOOHs: lipid hydroxiperoxide
MDA: malondialdehyde
4-HNE: 4-hydroxynonenal
APC: Adenomatous Polyposis Coli
NOC: N-nitroso compounds
ALA: 5-aminolevulinic acid
ALAS1: 5-Amilolevulinate Synthase 1
ALAD: ALA dehydratase
PBGD: porphobilinogen deaminase
UROS: uroporphyrinogen III synthase
UROD: uroporphyrinogen decarboxylase
CPOX: coproporphyrinogen oxidase
PPOX: protoporphyrinogen oxidase
FECH: ferrochelatase
PpIX: protoporphyrin IX
FGS: tumor fluorescence-guided surgery
PDT: photodynamic therapy
ETC: electron transport chain
OXPHOS: oxidative phosphorylation
ATP: adenosine triphosphate
HLRCC: hereditary leiomyomatosis and renal-cell cancer
TCA cycle: tricarboxylic acid cycle
ATPIF1: ATPase Inhibitory Factor 1
PDH: pyruvate dehydrogenase
ANT: adenine nucleotide transporter
GRBOX: glycolysis regulated box
TDO: tryptophan 2,3-dioxygenase
IDO1/2: indoleamine-2,3-dioxygenase 1 and 2
CYP1B1: cytochrome P450 family 1 subfamily B member 1
COX2: prostaglandin-endoperoxide synthase 2
NSCLC: non-small cell lung cancer
NR1D1 or Rev-erbα: nuclear receptor subfamily 1 group D member 1
NPAS2: neuronal PAS domain protein 2
PER2: period circadian regulator 2
HRM: heme-responsive motif
SLC46A1 or HCP1: solute carrier family 46 member 1
FLVCR2: feline leukemia virus subgroup C receptor family member 2
SLC48A1 or HRG1: solute carrier family 48 member 1
GLUT1: glucose transporter 1
FLVCR1: feline leukemia virus subgroup C receptor family member 1
ABCG2: ATP binding cassette subfamily G member 2
HER2: human epidermal growth factor receptor 2
TME: tumor microenvironment
TAMs: tumor-associated macrophages
RBCs: red blood cells
TECs: tumor-associated endothelial cells
ECM: extracellular matrix
MMPs: matrix metalloproteinases
CAFs: cancer-associated fibroblasts
MPO: myeloperoxidase
EPO: eosinophil peroxidase
NGF: nerve growth factor
DGCR8: DiGeorge critical region-8
miRNA: microRNA
CLOCK: clock circadian regulator
NCOR: nuclear receptor corepressor
HDAC3: histone deacetylase 3
GIS1: histone demethylase GIS1
jmjd-2/KDM4: lysine demethylase 4
AML: acute myeloid leukemia
CCs: cancer cells
Ac: acetylation
Me: methylation.
